# A randomized controlled trial testing the effectiveness of a universal school-based depression prevention program 'Op Volle Kracht' in the Netherlands

**DOI:** 10.1186/1471-2458-12-21

**Published:** 2012-01-10

**Authors:** Yuli R Tak, Rinka MP Van Zundert, Rowella CWM Kuijpers, Boukje S Van Vlokhoven, Hettie FW Rensink, Rutger CME Engels

**Affiliations:** 1 Behavioural Science Institute, Radboud University, Montessorilaan 3, 6525 HR Nijmegen, the Netherlands; 2 Trimbos Institute, Netherlands Institute of Mental Health and Addiction, Utrecht, the Netherlands

## Abstract

**Background:**

The incidence of depressive symptoms increases during adolescence, from 10.0% to 24.5% at age 11 to 15, respectively. Experiencing elevated levels of depressive symptoms increases the risk of a depressive disorder in adulthood. A universal school-based depression prevention program Op Volle Kracht (OVK) was developed, based on the Penn Resiliency Program, aimed at preventing the increase of depressive symptoms during adolescence and enhancing positive development. In this study the effectiveness of OVK will be tested and possible mediators of program effects will be focus of study as well.

**Method:**

The effectiveness of OVK will be tested in a randomized controlled trial with two conditions, intervention (OVK) and control condition (care as usual). Schools are randomly assigned to research conditions. OVK will be incorporated in the school curriculum, maximizing program attendance. OVK consists of 16 lessons of 50 min, given by trained psychologists to groups of 11-15 students. OVK contains Cognitive Behavioral Therapy, social skills training, problem solving and decision making. Outcomes are measured at 6, 12, 18 and 24 months follow up, to monitor long term program effects. Primary outcome is level of depressive symptoms, secondary outcomes are: anxiety, hopelessness, cognitive bias, substance use, truancy, life satisfaction, coping, self-efficacy, optimism, happiness, friendship, school performance and school attitude. The questionnaires for students will be administered in the school setting. Parents will complete a questionnaire at baseline only.

**Discussion:**

In this paper the study into the effectiveness of the depression prevention program OVK was described. It is expected that OVK will prevent the increase in depressive symptoms during adolescence and enhance positive development in the intervention condition, compared to the control condition. If OVK will be effective, it can be implemented in the school context by which numerous adolescents can be reached.

**Trial registration:**

Netherlands Trial Register (NTR): NTR2879

## Background

Depression is a significant public health concern that places a heavy burden on people and society. In the Netherlands, around 700.000 adults and 37.000 adolescents (13-17 years) are diagnosed with a depressive disorder annually [1]. During adolescence, the incidence of both depressive symptoms and disorder increases [2-5]. At ages 9 to 10, 0.5% of all children suffer from a depressive disorder, which rises to 3.4% at ages 15 to 16 [6]. At age 11, 10.0% experience depressive symptoms, at age 15 this has increased up to 24.5% [7]. Symptoms of depressive disorder involve for example: loss of interest in activities, weight and sleep problems, negative thoughts about the self, and thoughts about death and harming the self [8]. Across adolescence, girls are twice as likely as boys to report depressive symptoms and are at greater risk for developing a depressive disorder compared to boys [5,7]. The risk for an episode of depressive disorder in adulthood sharply increases when having experienced elevated symptoms of depression during adolescence [9], or when diagnosed with depressive disorder during adolescence [10]. Depressive disorder often co-exists with anxiety disorder, conduct disorder, oppositional deviant disorder, and is related to substance use [11]. In addition, depressive disorder is often associated with impairments in adolescents' school, family and social functioning [12]. Moreover, depressive disorder is associated with high treatment costs [1,13]. To conclude, there is a strong urge to prevent the onset of symptoms of depression and depressive disorder during adolescence.

Besides preventing the onset of aversive outcomes, adolescent functioning and development could be enhanced by promoting positive development. The Broaden and Build Theory posits that when experiencing positive emotions, people's thoughts and subsequent actions are expanded by becoming more creative and diverse when compared to experiencing negative emotions [14]. By means of these broadened thoughts and actions, people encounter more learning experiences, which in turn facilitate people's cognitive, social, physiological, and psychological development. In addition, experiencing positive emotions can undo the cardiovascular arousal negative emotions cause, as described in the Undoing Hypothesis [15]. It is therefore important to promote positive emotions in enhancing youth development. The importance of prevention and promotion of positive development in adolescence is also stressed by parents, teachers, and school principles. Research has shown that universal social emotional programs resulted in an improvement of academic performance, social and emotional skills, and in a reduction of conduct and internalizing problems [16].

Early adolescence offers great opportunities for mental health promotion and the prevention of mental health problems. As the brain matures, young adolescents' social, emotional and cognitive systems are developing [17,18], and the behavioral and cognitive systems develop at different rates [18]. On the one hand this poses challenges for optimal functioning in adolescents, but on the other hand it provides great opportunities for mental health promotion. Considering that adolescents have not fully matured cognitively and emotionally, adolescence is a period in which cognitive biases, reactions to stress, and self-efficacy beliefs are malleable. This is important given that self-efficacy, coping with stress, and cognitive biases are strongly related to depressive disorder and related characteristics [19-21]. Addressing self-efficacy, coping, and cognitive biases in depression prevention programs could promote youths' mental health and decrease the incidence of depressive symptoms and depressive disorder in adolescence.

Research shows that universal depression prevention programs can be effective in reducing depressive symptoms in adolescents [22,23]. Stronger effects are found in targeted prevention compared to universal prevention, but in targeted prevention not all adolescents at risk are identified [24]. In universal prevention the stigmatization effect associated with targeted prevention is no issue [25], and all adolescents are reached. Various universal depression prevention programs exist [22], but up till now, no universal depression prevention program has been empirically tested and implemented in the Netherlands. To fulfill this need, 'Op Volle Kracht' (OVK) was developed. OVK is a universal school-based depression prevention program for adolescents aged 12 to 14, that targets, among others, cognitive biases, coping skills and social skills. The theoretical basis of OVK is Cognitive-Behavioral Theory (CBT) of which the attendant therapy is known to effectively combat depression in adults [26], and is promising in treating depression in adolescents [27]. OVK is an adapted version of the Penn Resiliency Program (PRP), a U.S. depression prevention program that has shown to effectively prevent and decrease depressive symptoms in adolescents [28]. Important cultural and content-related modifications have been made to make the program suitable for Dutch teenagers.

The present study will test the effectiveness of OVK in an adolescent Dutch population by means of a randomized controlled trial with follow-up assessments up to 24 months. In addition, mechanisms by which the program effects will be obtained will by studied as well. Scholars have tested the effectiveness of depression prevention programs [22,23]. The mechanisms underlying program effects are still unknown. Identifying mediating factors can increase our understanding of resiliency development in youth, and could provide directions for improvements of prevention programs. In the prevention program 'Op Volle Kracht', different aspects are targeted that might function as mediating mechanisms in accomplishing prevention of depression. Research has shown that adolescents showing cognitive biases are at risk for developing a depressive disorder [21]. Also, avoidant coping styles [29,30], low social and academic self-efficacy [19], and hopelessness are significant predictors of depressive symptoms in adolescents [31]. Optimism has a preventive function, as higher levels of optimism predicted lower levels of depressive symptoms in adults [32]. Hence, improving adolescents' cognitions, coping, self-efficacy, hopefulness and optimism may prevent the onset of depressive symptoms in adolescents and foster resilience.

### Goals and hypotheses

The present study has three main goals: 1) Testing the effects of OVK in a Dutch adolescent population in limiting the increase of depressive symptoms during adolescence, 2) identifying mediating mechanisms that can explain program effects, and 3) testing the effects of OVK on secondary outcomes. It is hypothesized that OVK will be effective in preventing the increase in depressive symptoms in adolescence in the intervention condition during the 24 months following the program, in comparison to the control condition. In relation to identifying mediating mechanisms, it is hypothesized that OVK will lead to less cognitive biases, more adequate coping, higher self-efficacy, less hopelessness, and more optimism. Each of these constructs are hypothesized to lead to less (increase in) depressive symptoms; being mediators of the program effects on depressive symptoms. Secondary outcomes include anxiety, substance use, truancy, life satisfaction, happiness, friendship, school performance and school attitude.

## Methods

### Design

**Figure 1:**
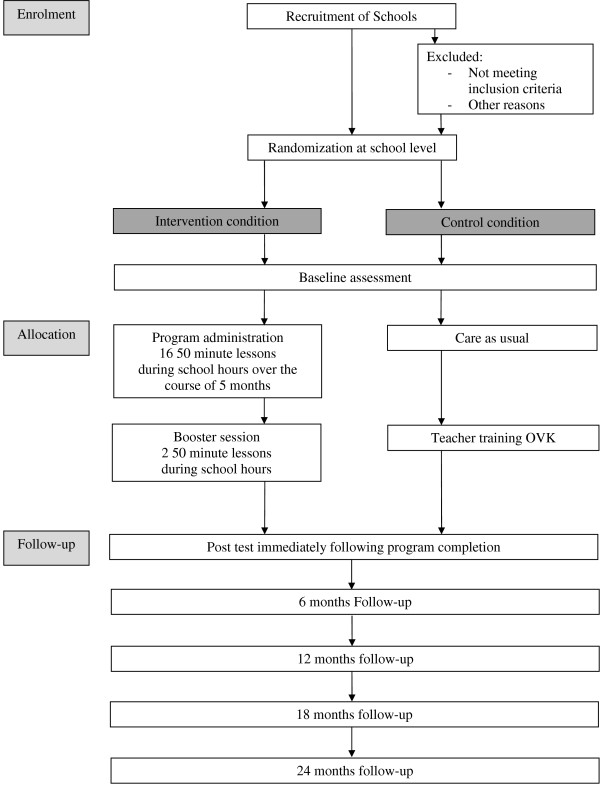
**Study design**.

The effectiveness of OVK will be studied by means of a randomized controlled trial with two conditions: intervention versus control (no intervention) (see Figure 1). We have chosen not to include a third condition with an alternative program, because there is no existing Dutch school-based universal depression prevention program for young adolescents, and because of financial constraints. Therefore, the control condition correctly reflects "care as usual". Randomization of schools, stratified by educational level, to either one of the conditions will be conducted by an independent statistician of the Radboud University. Stratifying by educational level is needed to evenly distribute educational level across intervention and control group, despite the small number of schools. Randomizing between schools (as opposed to within schools) will have the following benefits: It will minimizes contamination; adolescents in the control condition are not influenced by their friends in the experimental arm, and it will provide all adolescents in the second grade of a particular school with the opportunity to participate in the prevention program.

To test the (long term) effectiveness of the program, the outcomes will be assessed through self-report questionnaires approximately 1 month before the start of the program (baseline: T1), directly after the program (T2), and 6 (T3), 12 (T4), 18 (T5) and 24 (T6) months after the end of the program. Adolescents in the control condition will complete the questionnaires at exactly the same time points. Parental self-reports will be administered at baseline only. Adolescents assigned to the experimental condition will receive the OVK program, and adolescents in the control condition will receive care as usual (no program). As a reward for participation in the study, the schools in the control condition will receive a training for teachers in the OVK program between T3 and T4, on the strict condition that they will not provide the program to the adolescents who participated in the study.

OVK will be incorporated into the school curriculum; the 16-lesson program will be delivered once a week by trained psychologists to groups with a maximum of 15 adolescents. Incorporating the program in the school curriculum enables us to truly provide the program to all adolescents in a certain grade. As a result, high attendance rates will be assured and no stigmatization will occur. To keep the attrition rates at a minimum, all adolescents absent at a given measurement will be provided another possibility in the same time period to complete the questionnaire. Also, adolescents who leave school will be asked, with assistance of the school, to continue participating in the study. The trial is registered at the Dutch Trial Register: NTR2879.

### Participants and procedure

Participants will be adolescents in their second year of secondary school (8th grade, ages 12-14) [28]. Secondary schools in the southern and middle part of the Netherlands will be approached to participate in the study. Only adolescents in middle school grade 8 will be included. We will ensure inclusion of low SES and ethnic minority groups, as well as different educational levels (Dutch middle school prepares adolescents for vocational up to university level). The exclusion criterion consists of parental refusal of their child's participation in the study. When schools show interest, they will be asked to participate with as many as possible second grade classrooms. We expect that-once included in the study-across the entire study period, approximately 80% of adolescents will provide complete data [33,34].

A meta-analysis on 17 controlled studies on PRP indicated the significant average effect size to be 0.20 at 12-months follow-up with the CDI > 13 score as cut-off [28]. Power analysis (G-power) based on this 12-month mean effect size takes into account a maximum of 20% attrition over time, as well as clustering of data in schools and loss of power due to multiple imputation. Based on this power analysis, sample sizes need to be 662 adolescents per condition (alpha < .05, power = .80); at least 1,324 adolescents will be included.

With support from participating schools, parents of eligible adolescents will receive a letter, in which they will be fully informed about the study aims and procedure. For schools in the intervention condition a presentation will be held to inform parents and teachers about the content of the program and the study. Given that schools incorporate the program into their curriculum, passive consent will be obtained from parents. Each wave, adolescents will fill out a questionnaire in class during school hours. The 50-minute questionnaire will either be a web-based or a paper version, depending on the school's preferences. Parents fill out a 25-minute questionnaire at baseline only, addressing parental depression, anxiety, child and parental diagnostic status, and parenting skills. The mother will be asked to fill out the web-based questionnaire. Three weeks after the first mailing, non-responders will receive a reminder with the possibility to complete the questionnaire online or on paper. When not responding within 2 weeks, these parents will be contacted by phone. At any time, parents are free to withdraw themselves, and/or their child from the study. The Ethical Committee (ECG) of the Faculty of Social Sciences of the Radboud University Nijmegen has approved the study design and data collection.

### The program

#### Program theory

OVK is based on Beck's Cognitive Theory [35-37], the ABC-model of Ellis [38], and the Cognitive Theory of Seligman [39]. On the basis of their experiences, people consciously and unconsciously construct their own cognitive schemes by which they organize and process ideas and thoughts, feelings and experiences about the world, the future, other people and themselves [40]. The way people perceive the world and interpret their experiences depends upon the kind of cognitive schemes they have internalized. During a specific event, the cognitive scheme is activated, triggering an automatic thought that elicit a feeling. These automatic thoughts can either be positive, negative or neutral. In turn, these thoughts and feelings influence the way people behave, and the behavior displayed evokes certain consequences [40]. In CBT clients become aware of the link between thoughts, feelings and behavior, they learn to question the validity of automatic thoughts, to change negative automatic thoughts into more positive and realistic ones, to change their behavior, to cope with their problems, and to improve self-regulation [40].

#### Program content and structure

OVK consists of 16 lessons of 50 minutes. The first 8 lessons are devoted to explaining and practicing the CBT derived principles. Lessons 9 to 16 are directed at social and coping skills, self-esteem, problem solving, and decision making. During every lesson, the theory behind the techniques is shortly explained followed by actively practicing the skills by students. Several means are used for teaching, such as discussions, role-plays, and skits. Group leaders are responsible for group atmosphere and cohesion, and they will guide discussions and role-plays. Each lesson includes homework for the next lesson. Practicing skills facilitates internalization of these skills, which results in better program effects [23].

In lesson 1 to 8, students are familiarized with the CBT-principles; the relation between an event, thought, feeling, and behavior. First, students are introduced to the program, whereby building group cohesion, discussing confidentiality, and setting group rules are central. The students are told they will learn to deal with daily situations they feel or think pessimistically about. Afterwards, the link between a situation, thought, feeling and behavior is outlined. Inaccurate negative automatic thoughts (also called 'mind traps') are discussed. Challenging mind traps by means of formulating alternative realistic-optimistic thoughts on the spot, and searching for proof of your thoughts, is practiced. Lessons 9 to 16 are devoted to interpersonal problem-solving whereby the CBT-principles taught in the first half of the program are applied to the interpersonal domain. In sum, students learn about: (1) The link between situations, thoughts, feelings, and behaviors, 2) cognitive biases, including pessimistic versus optimistic explanatory styles, 3) cognitive restructuring skills, and 4) students learn a variety of techniques for coping and problem-solving, including assertiveness, negotiation, decision making, and relaxation.

#### Program delivery

The program will span approximately 5 months. Classes will be split up in two groups, thus consisting of 12-15 students, because this appeared to be the ideal class size for teaching PRP [28]. Gender constellation of the groups might differ. Previous research on all girl or co-ed groups does not show substantial differential effects of PRP on depressive symptoms as a function of gender constellation [41]. The 10 group leaders will have an MSc degree in clinical psychology or pedagogy. They will be trained during a 5-day workshop by two experienced psychologists of the Radboud University, who are both experts in Cognitive Behavioral Therapy. We will organize a group supervision session twice during the course of the program. Individual supervision will consist of frequent contact by phone and email to enable group leaders to feel supported, and to receive help when needed. Group leaders rate their adherence to the program content every lesson by completing a short questionnaire. The 10 group leaders will each deliver the program to 5-9 groups of students.

### Measures

The primary outcome will be the level of depressive symptoms at 6, 12, 18 and 24 months follow up measured with the Child Depression Inventory (CDI) [42], which is reliable and valid [43,44]. Possible mediators will be coping, cognitive bias, self-efficacy, hopelessness and optimism. The Children's Coping Strategies Checklist Revision 1 [45] will be used to assess coping styles. Cognitive bias will be measured with a questionnaire specifically developed to assess the cognitive biases addressed in OVK [46]. Self-efficacy will be assessed with the Self-Efficacy Questionnaire for Children SEQ-C [47], which has satisfactory internal consistency and good validity. Hopelessness will be measured with Beck's Hopelessness Scale (BHS) which has good reliability and validity [48]. Optimism will be measured with LOT-R which has good validity and reliability properties [49].

The 'negative' secondary outcomes will be the level of anxiety, truancy and substance use. Anxiety will be assessed with the Revised Children's Manifest Anxiety Scale (RCMAS) [50]. The RCMAS is a widely used questionnaire that assesses anxiety symptoms in children and adolescents. This scale has demonstrated good reliability and correlates with other measures of anxiety symptoms. Truancy will be measured through a self-report question: 'How many hours did you skip school the past 4 weeks?'. Widely used standard instruments will be administered to tap the frequency and intensity of alcohol [51,52] and tobacco use [53,54]. Positive secondary outcomes are life satisfaction, happiness, school grades and attitude, and participation in friendship groups. Life satisfaction will be measured with the Students Life Satisfaction Scale [55]. The SLSS assessed adolescents' global satisfaction or contentment. Studies have found the SLSS to have good internal consistency and test-retest reliability and to correlate highly with other subjective well-being measures [55]. Happiness will be measured with the Cantril Ladder [56]. Academic performance will be assessed by asking adolescents to list their last grades on core subjects. Clique membership and friendships will be measured with one sociometric item: "Who are your best friends?". Information on socio-demographic factors, for example, age, sex, ethnicity, religious affiliation, and educational level, will also be obtained.

### Parent measures

The parent questionnaire will include measures of socio-demographics of both parents and the participating child: Information about (prior) diagnosis and treatment, marital status, parental SES indicators such as education, employment, working hours, and salary. In addition, parents' own characteristics and parenting skills will be included (depressive symptoms, optimism, general parenting and mindful parenting). All instruments are widely used and have satisfactory psychometric properties. The Beck Depression Inventory [57] will be used to assess symptoms of depression. Anxiety will be tapped by the trait scale of STAI-DY; a Dutch version of the Spielberger State Trait Anxiety Inventory [58,59]. Optimism will be measured with the LOT-R [49]. General parenting, including responsiveness, and psychological and behavioral control, will be measured with the Dutch versions of the Acceptance-Rejection scale from the Child Report of Parent Behaviors Scale [60], 'Psychological Control' Scale-Youth Self-Report (PCS-YSR) [61], and the 'Parent Regulation Scale'-Youth Self-Report (PCS-YSR) [62], respectively. Parental autonomy support will be tapped with an adapted version of the Autonomy Support Scale [62,63]. Mindful parenting is assessed with the Dutch version of the Interpersonal Mindfulness Parenting Scale IM-P [64].

### Data analysis

Data will be analyzed in accordance with the intent-to-treat principle and for the completers only. Multiple imputations will be used for missing observations at follow-ups. The hypotheses will be tested with regression analyses for dichotomous outcome measures in MPLUS 5.1 [65,66]. Additional analyses will be conducted to test effects of the intervention on development of depressive symptoms using latent growth curve (LGC) modeling [66], in which the intercept and slope of levels of depressive symptoms using scale scores (as we will have 6 assessments) are modelled, and effects of condition can be examined. We will check for possible baseline differences between the two conditions in demographic variables (e.g. age, gender, school level, and ethnic background) and depressive symptoms. Moreover, variables that show different distributions between the two groups will be entered as confounders in all models testing the effectiveness of the program. The cluster effect-students were 'nested' in classes-will be handled by getting robust variance related estimates based on the first order Taylor series linearization method using Stata's procedures for design based analyses [65]. Reporting of the results of the study will be in accordance with the CONSORT statement [67].

## Discussion

In the present study the effectiveness of Op Volle Kracht, a universal school-based depression prevention program for adolescents, in preventing the increase in depressive symptoms during adolescence will be tested in a large sample (*N *= 1324) by means of a randomized controlled trial with an intervention and control condition. Follow-up measures will be conducted up to 24 months to capture long-term program effects. It is hypothesized that adolescents in the intervention condition will display less depressive symptoms during the follow-up measures, compared to adolescents in the control condition. It is expected that cognitive bias, problem and engagement-focused coping, self-efficacy, hopelessness, and optimism mediate the intervention effect on depression. Lastly, the present study will test whether OVK has effects on secondary 'negative' outcomes: Anxiety, substance use, and truancy, as well as on positive outcomes: Life satisfaction, happiness, friendship, and school performance and attitude.

### Strengths and limitations

Several strengths of the present study are identified. First, both prevention of aversive outcomes and the promotion of positive development are central in OVK. Therefore, program effects on depression, anxiety, and hopelessness, but also on optimism, happiness and life-satisfaction will be examined. Second, program effects will be tested in randomized controlled study and a large sample. Third, trained psychologists will administer the program [22,23]. Fourth, during OVK adolescents learn Cognitive Behavioral Therapy principles, which have been shown to be effective in treating depression in adults and adolescents [26,27]. Fifth, OVK is based on an effective universal depression prevention program [28]. Moreover, the program will be incorporated in the regular school curriculum, enabling all adolescents to participate in the program, maximizing high program attendance and minimizing measurement attrition rates.

Limitations of the present study are present as well. No placebo treatment will be included in the design, limiting the extent to which lower depressive symptoms in the intervention condition can be uniquely ascribed to the program. Another limitation of the study will be the single use of self-reports; interviews by trained professionals would be more sensitive in identifying depressive symptoms [68]. At the other hand, depressive symptoms can be measured reliably and validly by means of self-reports [43,44], and program effect sizes were not related to assessment mode [23]. Because of financial constraints, no integrity check performed by others than group leaders themselves will be conducted. Group leaders will fill out program adherence forms, enabling us to check which parts of each lesson were not covered and why.

### Implications for practice

If OVK will found to be effective in limiting the increase in depressive symptoms during adolescence over the 2-year follow-up period and by enhancing positive development, this will have positive consequences for the adolescents themselves, their family, friends, and for society. The program can be implemented in schools whereby the lives of adolescents can be enhanced and the negative consequences of experiencing depressive symptoms can be minimized. The needs expressed by school teachers and professionals to stimulate social and emotional development of adolescents at school are thereby addressed. Moreover, OVK has the potential to reach all adolescents without stigmatizing groups and without the risk of missing the ones who need the skills thought in OVK the most. In addition, the present study could increase our knowledge on the factors that contribute and foster intervention effects, thereby enabling us to understand the prevention of aversive outcomes and promotion of positive outcomes in adolescence, which could result in further improvement of universal depression prevention programs.

## Conclusion

The present paper described a Dutch adolescent school-based depression prevention program OVK and the study design into its effectiveness in preventing the onset of depressive symptoms and promoting positive outcomes over the 24 follow-up period. By means of this study a first attempt will be made to shed light on the mechanisms by which program effects will be obtained.

## Competing interests

The authors declare that they have no competing interests.

## Authors' contributions

YR is responsible for data collection, analysis, and reporting of study results. All others authors are supervisors and grant applicators. All authors read and approved the final protocol.

## Pre-publication history

The pre-publication history for this paper can be accessed here:

http://www.biomedcentral.com/1471-2458/12/21/prepub
